# Comparison of Perspectives on Cannabis Use Between Emergency Department Patients Who Are Users and Non-users

**DOI:** 10.5811/westjem.47368

**Published:** 2025-11-26

**Authors:** Catherine A. Marco, Lena Becker, Matthew Egner, Quincy Erturk, Ayush Sharma, Taylor Vail, Caroline Soderman, Nathan Morrison, Stephen Sandelich

**Affiliations:** * Penn State Health, Milton S. Hershey Medical Center, Department of Emergency Medicine, Hershey, Pennsylvania; † Penn State College of Medicine, Hershey, Pennsylvania

## Abstract

**Introduction:**

Many states have legalized the use of cannabis for medical or recreational purposes. Cannabis is commonly used both recreationally and medicinally, with therapeutic applications for conditions including chronic pain, seizure disorders, multiple sclerosis, anxiety, and depression. The purpose of this study was to compare emergency department (ED) patient knowledge of the short- and long-term effects of cannabis between users and non-users, and to understand perspectives and knowledge of cannabis use, to assist in development of public health interventions.

**Methods:**

We conducted this prospective survey study at Penn State Health – Milton S. Hershey Medical Center. Inclusion criteria included adult ED patients, ≥ 18 years of age, who had used cannabis in the most recent 30 days, between May to August 2024. The control group consisted of adult ED patients, ≥ 18 years of age, who had not used cannabis in the most recent 30 days. We conducted thematic analysis to identify subjects’ knowledge of positive and negative effects of cannabis use.

**Results:**

Of 258 eligible subjects, 169 consented to participate (65.5%). Most identified as female (54.4%) and White (68.1%), with a mean age of 40 years. Most participants reported cannabis use in their lifetime (75.7%). Participants reported a myriad of reasons for using cannabis, including to treat anxiety (N = 67; 40%); pain (N = 65; 38%); recreational use (N = 62; 37%); sleep (N = 48; 28%); and depression (N = 34; 20%). Commonly perceived positive effects of cannabis use included relaxation (18%), pain relief (16%), and improved mental health symptoms (13%). Commonly perceived negative effects of cannabis use included cognitive impairment (11%), addictive potential (7%), pulmonary effects (8%), and worsened mental health symptoms (6%). Cannabis users were less likely to correctly identify negative short-term and long-term consequences of cannabis use, compared to non-users. Cannabis users scored mean 2.51/5 (95% CI 2.11–2.92) for correctness of negative short-term effects, compared to 3.28/5 (95% CI 2.96–3.6) for non-users (*P* = .004). Cannabis users scored mean 1.78/5 (95% CI 1.44–2.12) for correctness of negative long-term effects, compared to 2.38/5 (95% CI 2–2.76) for non-users (*P* = .002).

**Conclusion:**

Among ED patients who reported using cannabis, reasons cited for its use included recreation, anxiety, pain, depression, and sleep. Emergency department patients had significant knowledge gaps regarding the effects of cannabis use, and these knowledge gaps were higher among cannabis users. Cannabis users were less likely to correctly identify negative short-term and long-term consequences of cannabis use, compared to non-users.

## INTRODUCTION

Cannabis is a drug derived from the cannabis plant, with delta-9-tetrahydrocannabinol (THC) being its primary psychoactive component, which produces its effects in humans and animals. As one of the most widely used substances globally, approximately 45% of United States residents have tried cannabis at least once in their lifetime.^1^ In the 1990s, Simons et al categorized five motives for cannabis use: enhancement; conformity; mind expansion; coping; and social motives.^2^ Cannabis is commonly used both recreationally and medicinally, with therapeutic applications for conditions including chronic pain, seizure disorders, multiple sclerosis, anxiety, and depression. In chronic pain users, cannabis has been shown to reduce pain, improve sleep, and reduce anxiety and depression.^3^

The literature is mixed on the effects of cannabis. Short-term effects of cannabis can include attention impairment, increased impulsivity, and impairments in working memory and decision-making, with these effects being more common in irregular users. Chronic, frequent users often experience long-term effects such as a slower processing speed, impaired decision-making, and impaired attention when abstinent from cannabis. Long-term effects include airway inflammation, cardiovascular effects, including tachycardia and increasing the risk of myocardial infarction.^4^,^5^ With the growing legalization of cannabis, its usage is increasing.^6^,^7^ Despite the common perception that there are no risks with cannabis, approximately 10% of users will develop dependence.^8^

There has also been a growing rate of cannabis-related emergency department (ED) visits over the last two decades. In addition to psychiatric complaints, the most common reasons for this population to visit the ED are intoxication and gastrointestinal (GI) symptoms. Cannabinoid hyperemesis syndrome (CHS) is a disorder characterized by cyclic nausea and vomiting with daily cannabis use. Habboushe et al found that 32.9% of near-daily or daily users who came to the ED for a non-GI complaint also had symptoms of CHS.^9^ Although more prevalent, GI and psychiatric complaints are less likely to result in hospital admission. Patients with more rare complaints, including dermatologic, respiratory, trauma, and cardiovascular, are more likely to be admitted.^10^

Previous studies have explored perceptions of cannabis and its effects. Fennell et al found that patients who use cannabis for chronic pain perceive their use as having low risks and moderate benefits. Those who did perceive risks were more likely to have poorer mental health and more problems with cannabis.^11^ Marco et al found significant discordance between patient and physician perspectives on the clinical effects of cannabis.^1^

Our goal in this study was to explore ED patient knowledge of the short- and long-term effects of cannabis use among cannabis users and non-users. By understanding both the knowledge and perspectives of those who use cannabis, we aimed to compare ED patient understanding of the short- and long-term effects of cannabis between users and non-users, and to build on these perspectives to assist in development of public health interventions.

**Population Health Research Capsule**

What do we already know about this issue?

*Previous studies have explored perceptions of cannabis and its effects. There is significant discordance between patient and physician perspectives on its clinical effects*.

What was the research question?

*We compared emergency department (ED) patient knowledge of the short- and long-term effects of cannabis between users and non-users*.

What was the major finding of the study?

*Cannabis users scored mean 2.51/5 (95% CI 2.11–2.92) for correctness of negative short-term effects, compared to 3.28/5 (95% CI 2.96–3.6) for non-users (P = .004)*.

How does this improve population health?

*The significant knowledge gaps regarding effects of cannabis use represents an opportunity for patient education*.

## METHODS

This cross-sectional survey study was approved by the Penn State University Institutional Review Board (STUDY00024608). Eligible subjects were recruited as a convenience sample when a research assistant (RA) was available, which included all days of the week, and hours between 8 am–12 am. Inclusion criteria included adult patients in the Milton S. Hershey Medical Center ED, ≥ 18 years of age, who had used cannabis in the most recent 30 day between May–August 2024; for purposes of this study, they were categorized as cannabis users. Adult ED patients ≥ 18 years of age who had not used cannabis in the most recent 30 days comprised the control group entitled non-users (terminology cited in previous published literature).^12^–^13^ Subjects were excluded if they did not speak English, were prisoners, or were in distress, as defined by requiring resuscitation or unable to participate. Written informed consent was obtained. A total of six trained RAs administered the survey verbally without prompts and recorded subject responses.

The survey was developed by researchers, based on a previously published study instrument,^14^ and it was pilot-tested among a focus group of ED patients for clarity. Data collected included the study participants’ demographic information, personal cannabis use habits, and their awareness of the legality of cannabis use and their reported understanding of positive and negative short-term and long-term effects of cannabis. Open-ended questions allowed participants to describe positive and negative effects of cannabis in their own words to avoid interpretation bias. (Appendix A).

We conducted thematic analysis using MAXQDA v24.6.0 (VERBI Software GmbH, Berlin, Germany) to systematically identify and analyze themes from participants’ responses to open-ended questions regarding cannabis use.^15^ The process followed a rigorous approach to ensure reliability and validity, as described below. The codebook was developed through inductive coding (Appendix B). Two coders applied the codes to a 20% validation dataset to establish interrater reliability (IRR); the Cohen kappa (κ) was then calculated. Discrepancies in coding were flagged by the software and reviewed by a third coder, who adjudicated disagreements to achieve consensus. After coding, we analyzed themes using MAXQDA’s visualization and reporting tools. Frequencies and co-occurrences of codes were examined to identify dominant patterns.

Following thematic analysis, we coded responses for accuracy. Correct responses were defined a priori, based on three authoritative sources (Table 1).^16^–^18^ Three reviewers—one EM faculty, one pharmacology faculty, and one EM resident who were blinded to the study hypothesis and to the cannabis use of the participant—scored correct responses using a modified Likert scale of 0–5 (0 = completely incorrect; 5 = completely correct), to allow partial credit for variable understanding of the effects of cannabis. We used parametric statistics (the Student *t*-test) to compare groups by their Likert scale responses due to relatively large sample size, where the differences between results of parametric and non-parametric comparisons are largely inconsequential. A statistician analyzed data using SAS software v9.4 (SAS Institute Inc, Cary, NC). Descriptive data were expressed using frequency and percent. Comparison between groups was performed using the Fisher exact test.

## RESULTS

### Characteristics of Study Participants

Of 258 eligible subjects, 169 consented to participate (65.5%). Most identified as female (54.4%) and White (68.1%), with a mean age of 40 years. Demographic data is described in Table 2. Patients were interviewed each day of the week, with Thursday being the most common (20.7%). Most participants (82.9%) arrived via walk-in, and all were triaged as either an Emergency Severity Index (ESI) level 2, 3, or 4.

### Cannabis and Other Substance-use Habits

Most participants reported cannabis use in their lifetime (75.7%). The average number of days of cannabis use in the prior month across all participants was 11.6, with a wide variation in number of individual uses in the past month, 0 (minimum) – 1,500 (maximum). Participants who reported cannabis use had used it for mean nine years and first used cannabis at mean age 20. Participants reported a myriad of reasons for using cannabis, including to treat anxiety (N = 67; 40% pain (N = 65; 38%); recreational use (N = 62; 37%); sleep (N = 48; 28%); and depression (N = 34; 20%). Thirty percent of participants reported current possession of a medical cannabis prescription. Over 95% of participants reported no recreational drug use outside cannabis. Alcohol use was reported by 51.2% of participants, citing either drinking alcohol daily or socially.

### Participant Knowledge of State Cannabis Legislation

Of the participants, 163 resided in Pennsylvania, where the study was conducted, while six participants resided out of state. Most participants (N = 147; 87%) correctly answered questions about legality of cannabis in their home state.[Fig f1-wjem-26-1598]

### Knowledge of Effects of Cannabis

We analyzed five open-ended questions focusing on the short- and long-term consequences of cannabis use, both positive and negative. Responses were collected from 169 participants, yielding a total of 676 individual responses across all questions. Participants were able to identify multiple positive and negative short- and long-term effects. The IRR was found to be acceptable with a κ value of 0.87 (Table 3).

### Consequences of Short-term Cannabis Use

#### Positive Consequences

Among participants, 97.0% identified at least one short-term positive effect of cannabis use. The most commonly mentioned benefits included relaxation and calming effects (18.2%), pain relief (16.3%), and improved mental health symptoms (12.7%). Additionally, cannabis was noted for increasing appetite (8.1%), aiding in sleep (8.1%), and enhancing mental focus and cognition (3.3%).

#### Negative Consequences

Short-term negative effects were reported by 91.1% of participants. These included cognitive impairments such as brain fog (10.7%), physical effects like cotton mouth or lung irritation (7.8%), and worsened mental health symptoms (5.5%). Participants also highlighted concerns about diminished reaction time and driving impairment (4.3%).[Fig f2-wjem-26-1598]

### Long-term Consequences

#### Positive Consequences

With regard to long-term cannabis use, 90.5% of participants described positive outcomes. Key benefits included chronic pain relief (7.5%), improved mental health symptoms (8.0%), and therapeutic uses for conditions such as cancer (1.8%). Other benefits, such as increased appetite and weight gain, were noted less frequently (0.6%).

#### Negative Consequences

Long-term negative consequences were reported by 84.6% of respondents. Dependency or addictive potential was highlighted by 7.0%, with negative pulmonary effects (5.9%) and worsened mental health symptoms (5.5%) also commonly mentioned. Concerns regarding cost (0.7%) and driving impairment (0.7%) were noted less often but remain relevant.

#### Differences Between Cannabis Users and Non-Users

At the time of interview, 74 participants were classified as current cannabis users and 86 as non-users. Nine participants were not classified as either user or non-users. Those classified as cannabis non-users (μ = 43.6 years of age) were significantly older than cannabis users (μ = 36.5 years of age, *P* = .03), although their sex and ethnicity were not significantly different. Cannabis users were more likely to answer correctly about the legality of cannabis for recreational use in the state where they lived (94.19%, χ^2^ = 0.0190, *P* = .02). There was no significant difference between cannabis users’ and non-users’ answers regarding the legality of cannabis use for medical purposes in the state where they lived (χ^2^= 0.1718, *P* = .25).

#### Cannabis Users’ and Non-Users’ Perceptions of the Effects of Cannabis

**Figure 3:**
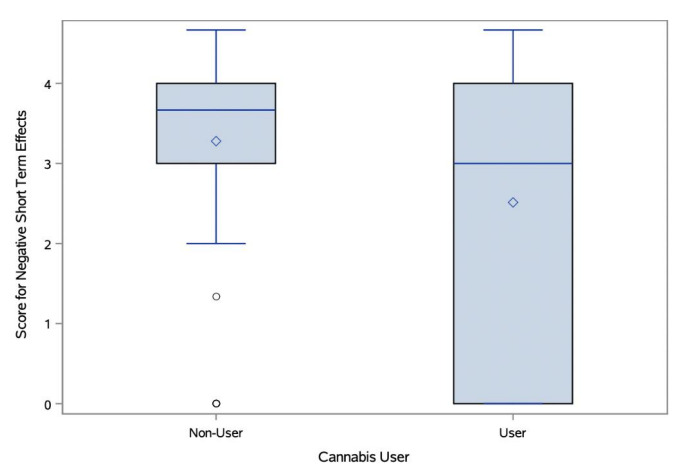
Negative short-term effects response scores by cannabis users compared to non-users.

**Figure 4:**
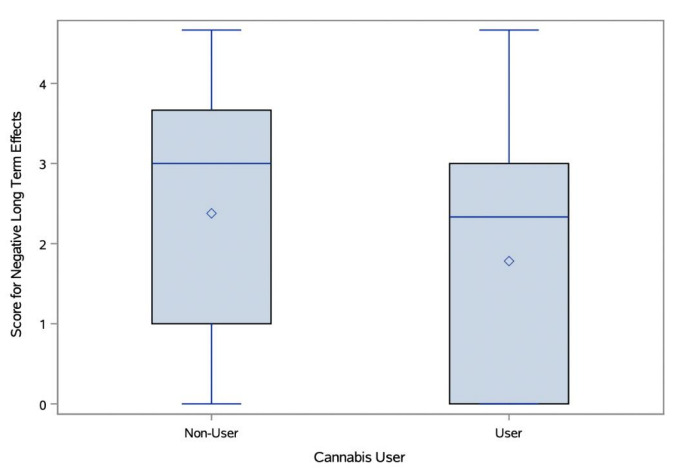
Negative long-term effects response scores by cannabis users compared to non-users.

Participant answers to open-ended questions about the positive and negative short- and long-term effects of cannabis use were evaluated by three blinded scorers. The IRR ranged from moderate to excellent agreement (K = 0.56–0.83). Cannabis users and non-users displayed statistically significant differences in the correctness of their responses about the negative short- and long-term effects of cannabis use, with non-users rated as more correct in their responses (Figure 3 and Figure 4). Cannabis users scored mean 2.51/5 (95% CI 2.11–2.92) for correctness of negative short-term effects, while non-users scored a mean 3.28/5 (95% CI 2.96–3.6; *t*-test *P* = .004). Cannabis users scored a mean 1.78/5 (95% CI 1.44–2.12) for correctness of negative long-term effects, while non-users scored a mean 2.38/5 (95% CI 2–2.76; *t*-test *P* = .002). There were no differences between groups in correctness of perception about positive effects, either short- or long-term.

## DISCUSSION

In this study, cannabis non-users reported higher numbers of negative short- and long-term effects compared to cannabis users. Most participants (83%) of correctly stated that recreational cannabis is illegal in the state of Pennsylvania.

Effects of cannabis on cognitive and physiologic function have been previously reported. Cannabis use is associated with impaired neurophysiological function regarding memory, learning, attention, coordination, emotions, and reaction time.^19^,^20^ Chronic cannabis use at a young age (< 18 years) has been found to disrupt normal brain development. Acute cannabis intoxication has the potential to impact working and episodic memory, behavioral disinhibition, and impulsivity; in the *Coronary Artery Risk Development in Young Adults* study, chronic exposure to cannabis was associated with worse verbal memory.^21^ Additionally, approximately 30% of cannabis users will develop cannabis use disorder. With an increasing incidence of mental health illnesses in the US, close attention is being paid to a connection with cannabis usage.

Cannabis use is thought to be a preventable risk factor for the development of psychosis. In one investigation, daily cannabis use was associated with at 3.2 times greater odds of developing a psychotic disorder.^22^ Among many studies, long-term heavy cannabis use was associated with underachievement and impaired motivation. Interestingly, one study found an association between suicidality trends from 2008–2019 and cannabis use, although the researchers identified potential overlapping risk factors and cited the need for further research.^23^ Cannabis use is associated with motor vehicle crashes, malignancy, negative cardiovascular outcomes, and ED visits. Regular smoking of cannabis is associated with airway inflammation similar to cigarette smoking. Although no link has been established between cannabis use and cancer, research is ongoing. Cannabinoid hyperemesis syndrome is associated with weekly use of cannabis. Characterized by several cyclic vomiting episodes accompanied by abdominal pain, CHS may be due to derangements in the body’s intrinsic control of nausea and vomiting from chronic overstimulation of endocannabinoid receptors. Further complications of this syndrome include acid/base abnormalities and aspiration pneumonitis.^24^

A recent study cited significant adverse health effects associated with legalization of recreational cannabis use, including psychosis, suicide, other substance use, increases in the number of fatal motor vehicle collisions, and others.^25^ Another study made conclusions regarding heart disease, vascular accidents, and cannabis use. Cannabis is now emerging as a predictor or potential risk factor for heart failure and cerebrovascular accidents.^26^ Jeffers et al recently published a study controlling for tobacco smoking when studying cannabis use and cardiovascular outcomes. Using a behavioral risk factor surveillance survey from 27 states in the US, the group suggested in their cross-sectional study that cannabis use is associated with higher risk of myocardial infarction and stroke.^27^

Visits to the ED involving cannabis use have increased 12–17% per annum over the past few decades.^28^ The US Centers for Disease Control and Prevention (CDC) used national surveillance program data to study cannabis-related ED visits during the COVID-19 pandemic in 2020–2022 for people < 25 years of age. This analysis found that the largest increase in cannabis-related ED visits was in children ≤ 10 years of age from pre-pandemic to pandemic times.^29^

We found significant knowledge gaps among ED patients regarding cannabis use. Many patients, particularly cannabis users, are unaware of negative effects of cannabis use. As more US residents use cannabis as a recreational activity and as a medical therapy, informing and educating the public about the risks regarding cannabis, similar to alcohol and tobacco use prevention campaigns, is crucial. Future directions include public health measures to identify and correct knowledge gaps regarding safety of cannabis use and potential negative effects of cannabis use. Future research should also identify and quantify negative physiologic and cognitive effects to better inform safe medical use or avoidance of cannabis use.

## LIMITATIONS

Among the limitations of this study was its small sample size of 169 participants. Additionally, while the population demographics did match typical Pennsylvania demographics, this is specific to the state and may not be generalizable to the entire country. Further, the survey instrument was original and has not been validated. It should be noted that the number of eligible subjects was based on RA recall and that data were collected based on RA availability. Finally, because data were largely self-reported by participants, this could have led to potential bias, inaccuracy, or survey fatigue.

## CONCLUSION

Among ED patients who used cannabis, reasons cited for cannabis use included recreation, anxiety, pain, depression, and sleep. The study participants had significant knowledge gaps regarding effects of cannabis use, and these knowledge gaps were higher among cannabis users. Cannabis users were less likely to correctly identify negative short-term and long-term consequences of cannabis use, compared to non-users.

## Supplementary Information





## Figures and Tables

**Figure 1 f1-wjem-26-1598:**
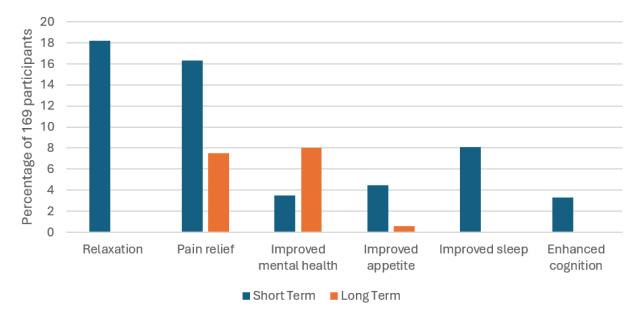
Positive effects of cannabis as reported by 169 participants.

**Figure 2 f2-wjem-26-1598:**
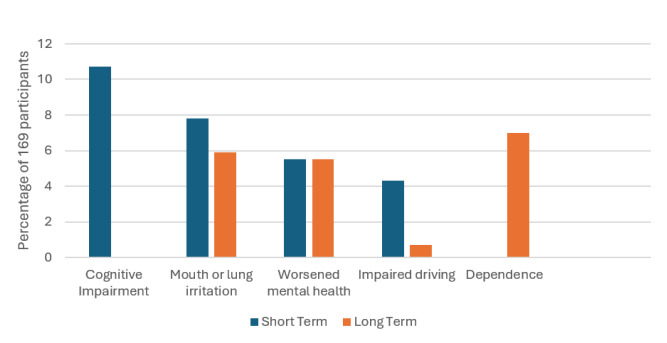
Negative effects of cannabis as reported by 169 participants.

**Table 1:** Short- and long-term effects of cannabis use.^16^,^17^,^18^

Clinical Category	Short-term effects of cannabis use	Long-term effects of cannabis use
Mental Health	Euphoria, relaxation, sedation, hallucinations	Anxiety, depression, suicidal thoughts, sleep disorder, psychosis, delirium, schizophrenia, risk of substance use disorder
Gastrointestinal	Increased appetite, dry mouth	Cannabinoid hyperemesis syndrome
Neurologic	Impaired short-term memory, impaired concentration, impaired psychomotor coordination, slurred speech	Impaired cognition, reduced frequency of seizures
Cardiovascular	Tachycardia, hypertension, arrhythmia	Increased risk of stroke, heart disease, and other vascular diseases Pediatrics: impaired attention, memory, and learning
Other	Impaired driving skills (including slowed reaction time and ability to make decisions, impairing coordination, and distorting perception), impaired time perception	Reduction of chronic pain, reduction of musculoskeletal pain, low birth weight, NICU admission

*NICU*, neonatal intensive care unit.

**Table 2:** Demographic Information of 169 participants in study of the effects of cannabis use.

	n (%)	Users	Non-users
Sex			
Male	77 (45.6)	45 (61.6%)	40 (41.7%)
Female	92 (54.4)	28 (38.3%)	56 (58.3%)
Race/Ethnicity			
Black	16 (9.5)	13 (13.6%)	4 (5%)
Asian	2 (1.2)	2 (2%)	0
Hispanic	20 (11.8)	11 (11.4%)	9 (12.3%)
Multiracial	16 (9.5)	11 (11.4%)	5 (6.8%)
White	115 (68.1)	59 (61.5%)	55 (75.3%)
Age *M* (SD)	40.4 (14.6)	38	44

**Table 3:** Results of thematic analysis of open-ended questions regarding positive and negative short- and long-term effects of cannabis use among 169 participants.

	Frequency	Percentage
Positive Short-term	656	97.0
Relaxation/Calms mood	123	18.2
Pain relief	110	16.3
Increases appetite	55	8.1
Helps with sleep	55	8.1
Improves mental health	54	8
Improves cognition	22	3.3
Fun/Social	11	1.6
Negative Short-term	616	91.1
Lazy, sleepy, brain fog	72	10.1
Physical body effects	53	7.8
Worsens mental health	37	5.5
Diminished reaction/driving	29	4.3
Increased appetite	29	4.3
Addictive potential	19	2.8
Cost	5	0.7
Positive Long-term	612	90.5
Mental health	86	12.7
Pain	51	7.5
Addiction	12	1.8
Cancer	12	1.8
Appetite	12	1.8
Negative Long-term	572	84.6
Negative brain effects	53	7.8
Addictive effect	47	7.0
Negative lung effects	40	5.9
Impaired driving	5	0.7
Increased appetite	4	0.6
