# Supplementary material for: Comparison of Perspectives on Cannabis Use Between Emergency Department Patients Who Are Users and Non-users
Source: West J Emerg Med. 2025 Nov 26;26(6):1598–604. doi: 10.5811/westjem.47368 (PMC12698149; doi:10.5811/westjem.47368)
Supplement: Supplementary file 1 [file wjem-26-1598-s001.docx]

Effects of Cannabis Use: Perspectives of Emergency Department Patients

Survey Instrument

**STUDY ID _______ (Research assistant initials and consecutive numbers; NO PHI)**

Day of the week:

___(1) Sun ___(2) Mon

___(3) Tues ___(4) Wed

___(5) Thurs ___(6) Fri

___(7) Sat

Patient age (years) ________ (if 90 or older, enter “90”)

Patient gender

___(1) Male

___(2) Female

Patient ethnicity

__(1) African American

__(2) Asian

__(3) White

__(4) Hispanic

__(5) Multiracial

__(6) Other _____________________________________

Mode of Arrival in ED

___(1) Walk-In

___(2) Ambulance

ED Chief Complaint____________________________________(enter free text)

Triage level 1 2 3 4 5

(circle one)

Patient Survey

1. Have you ever used marijuana or cannabis? Y N

2. In the last 30 days, how many days have you used marijuana or cannabis?

In the last 30 days, how many times have you used marijuana or cannabis?

3. In your lifetime, how many years (or months) have you used marijuana or cannabis?

4. When did you first use marijuana or cannabis? (Age)______________

5. When was your most recent use of marijuana or cannabis?

(T) Today

(Y)Yesterday

(W)Within the past week

(M)Within the past month

(A)1-12 months ago

___years ago

6. What route do you use marijuana or cannabis?

___(S)Smoke

___(V) Vape

___(E)Edible (includes food, tea, etc.)

___Other_________________

6. What are the reasons you use marijuana or cannabis? (select all that apply)

___(R)recreational

___(P)to treat pain

___(D)to treat depression

___(A)to treat anxiety

___(S)to treat sleep disorder

___other_________________________

7. Do you have a prescription for marijuana? ___yes ___no

8. Have you used any other street drugs in the past 30 days?

(O) opiates

(C) cocaine

(M) methamphetamine

(B) benzodiazepine

(O) other____________________

9. How often do you drink alcohol?

(D) daily

(S) socially

(N) never

10. What state do you reside in?

11. In your state, is recreational cannabis legal?

___(Y)Yes ___(N)No ___(U)Unsure

12. In your state, is prescribed cannabis (“medical marijuana”) legal?

___(Y)Yes ___(N)No ___(U)Unsure

14. What are the short term effects of cannabis use (within 24 hours)? Please list both positive and negative effects.

15. What are the long term effects of cannabis use (after 24 hours)? Please list both positive and negative effects.

ED Diagnosis____________________________________

ED Disposition _________ (D)Discharge to home

_________ (H)Hospital admission

_________ (P)Admission to psychiatric facility
