# Supplementary material for: Comparison of Perspectives on Cannabis Use Between Emergency Department Patients Who Are Users and Non-users
Source: West J Emerg Med. 2025 Nov 26;26(6):1598–604. doi: 10.5811/westjem.47368 (PMC12698149; doi:10.5811/westjem.47368)
Supplement: Supplementary file 2 [file wjem-26-1598-s002.docx]

Codebook: Thematic Analysis of Open-Ended Questions

|  | Frequency | Percentage |
| --- | --- | --- |
| **Positive Short Term** | **656** | **97.0** |
| Relaxation/Calms Mood | 123 | 18.2 |
| Pain Relief | 110 | 16.3 |
| Increases Appetite | 55 | 8.1 |
| Helps with Sleep | 55 | 8.1 |
| Improves Mental Health | 54 | 8 |
| Improves Cognition | 22 | 3.3 |
| Fun/Social | 11 | 1.6 |
| **Negative Short Term** | **616** | **91.1** |
| Lazy, sleepy, brain fog | 72 | 10.1 |
| Physical body effects | 53 | 7.8 |
| Worsens mental health | 37 | 5.5 |
| Diminished reaction/driving | 29 | 4.3 |
| Increased appetite | 29 | 4.3 |
| Addictive potential | 19 | 2.8 |
| Cost | 5 | 0.7 |
| **Positive Long Term** | **612** | **90.5** |
| Mental Health | 86 | 12.7 |
| Pain | 51 | 7.5 |
| Addiction | 12 | 1.8 |
| Cancer | 12 | 1.8 |
| Appetite | 12 | 1.8 |
| **Negative Long Term** | 572 | 84.6 |
| Negative brain effects | 53 | 7.8 |
| Addictive effect | 47 | 7.0 |
| Negative lung effects | 40 | 5.9 |
| Impaired driving | 5 | 0.7 |
| Increased appetite== | 4 | 0.6 |
